# A New Image Grating Sensor for Linear Displacement Measurement and Its Error Analysis

**DOI:** 10.3390/s22124361

**Published:** 2022-06-09

**Authors:** Fang Cheng, Dongfang Zhou, Qing Yu, Tegoeh Tjahjowidodo

**Affiliations:** 1 College of Mechanical Engineering and Automation, Huaqiao University, Xiamen 361021, China; zhoudf@stu.hqu.edu.cn (D.Z.); yuqing@hqu.edu.cn (Q.Y.); 2 Department of Mechanical Engineering, De Nayer Campus, KU Leuven, Jan Pieter de Nayerlaan 5, 2860 Sint-Katelijne-Waver, Belgium; tegoeh.tjahjowidodo@kuleuven.be

**Keywords:** displacement measurement, image grating, vision-based, phase correlation, range expansion, error analysis

## Abstract

To improve the accuracy of the current vision-based linear displacement measurement in a large range, a new type of linear displacement sensing system, namely, image grating, is proposed in this paper. The proposed system included a patterned glass plate attached to the moving object and an ultra-low distortion lens for high-accuracy image matching. A DFT local up-sampling phase correlation method was adopted to obtain the sub-pixel translation of the patterns onto the target plate. Multiple sets of stripe patterns with different designs were located on the glass plate to expand the measurement range, based on the principle of phase correlation. In order to improve the measurement accuracy, the main errors of the image grating system were analyzed, and the nonlinear error compensation was completed based on the dynamic calibration of the pixel equivalent. The measurement results, after the error compensation, showed that the total error of the proposed system was less than 2.5 μm in the range of 60 mm, and the repeatability was within 0.16 μm, as quantified by standard deviation.

## 1. Introduction

Displacement measurement plays a very important role in various industries. Common displacement sensors include Hall sensors [1], fiber sensors [2,3], interferometric sensors, grating-based sensors, etc. With the rapid development of advanced manufacturing, high-precision displacement sensing techniques over a long range are in high demand. Typical applications include the processing and measurement of large-size wafers [4], positioning and process control of CNC machine tools [5], development of coordinate measuring machines [6], etc. At present, most high-precision, large-range displacement measurement systems are developed by using laser interferometry or traditional grating sensing. Laser interferometers have the highest accuracy, but they require precise alignment installation and very strict environmental control, which limits their application in industry [7,8]. Traditional grating displacement sensors include optical encoders, magnetic encoders, capacitive encoders and the inductosyn. The resolution of these sensors depends on the grating pitch, and there are obvious cumulative errors. In addition, they all adopt a structure where the scale and the reading head are close to each other, and the short stand-off distance limits their installation flexibility, which creates certain difficulties in the optimization of the instrument structure [9]. For brownfield applications, where the available manufacturing process does not allow disruptive updates or the replacement of existing processes due to the constraints of time, cost or footprint, visual sensors with fast and flexible installation are needed.

Therefore, visual measurement technology has been progressing rapidly in recent years. With the advantages of modular design, low cost, non-contact, long stand-off, and high resolution, it has been successfully applied in 3D reconstruction [10,11], target detection [12], deformation analysis [13,14], etc. For long-travel measurement, a linear displacement measurement method based on image grayscale information was developed by mapping the grayscale values of each pixel on a periodic pattern [15]. Experimental results showed that the standard deviation was 4 μm within the measurement range of 10 mm.

The image correlation method is a common method for studying in-plane deformation or rigid body displacement [16,17]. Using sub-pixel registration makes it possible to achieve submicron resolution. In order to counteract the limitation of the measurement range of the periodic stripe pattern, a target image of double periodic stripe pattern was proposed, but the range improvement was not significant [18]. Another approach was to apply a camera that moved with the stage to take images with a non-periodic sinusoidal stripe pattern for motion tracking. Meanwhile, various sub-pixel interpolation algorithms based on phase correlation were proposed, and the speed, accuracy and robustness of these algorithms were compared and analyzed [19,20,21]. In the field of micro-scale measurement, a pseudo-periodic pattern was proposed to determine the unwrapped phase value for a 3-DOF position measurement in the plane. Its range-to-resolution ratio was proven to be 10^8^ [22].

These linear displacement measurement methods based on vision have their own merits, but the challenge of high-precision measurement in a large range have not been well addressed. One of the key error factors, the optical model’s imperfection, which significantly affects the displacement measurement accuracy, has hardly been discussed.

In order to address this challenge, an image grating technique was proposed based on an optical distortion correction model [23]. By compensating for the optical distortion over the whole field of view (FoV), the image grating system was able to achieve submicron accuracy in a measurement range of 50 mm.

The work proposed in this paper is a continuation of the work published in [23]. To further enlarge the measurement range and to compensate for the measurement errors induced by the range expansion were the main objectives of this study. The proposed system increased the installation distance between the displacement reader and the scale through the lens, and it can achieve high measurement accuracy in a larger range. Further details are provided in the following sections.

## 2. The Principle of the Measurement System

### 2.1. The Working Principle of the Measurement System

**Figure 1:**
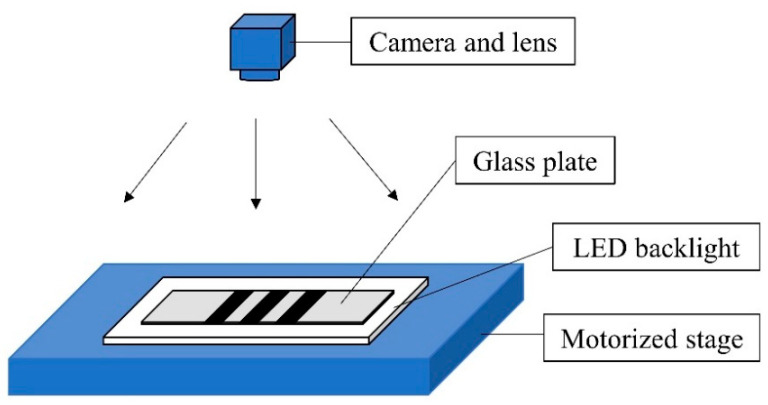
Schematic diagram of the working principle of the measurement system.

The working principle of the proposed image grating system is shown in Figure 1. The glass plate is placed above the LED backlight. The LED light passes through the glass plate to generate black and white stripes. When the stage moves, images with stripe patterns at different positions are captured by the camera. By performing phase correlation on the images before and after the translation, the number of pixels that shift is obtained, and then the displacement of the stage can be acquired by the proportional relationship between the object and image.

### 2.2. DFT Local Up-Sampling Phase Correlation Method

In order to obtain the sub-pixel translation of the target features quickly and accurately, A DFT local up-sampling phase correlation method was adopted in this study [23,24,25]. It can be divided into two steps: whole-pixel positioning and sub-pixel positioning. Since the proposed system only involved one-dimensional displacement, only the one-dimensional case was considered.

The first step was the whole-pixel positioning. The initial position image in the space was g(x), and the translated image was $f\left(x\right)=g\left(x-x_{0}\right)$. Fourier transforms of f(x) and g(x) were taken to obtain F(u) and G(u), respectively, and then phase changes representing the pattern translation were obtained through the normalized cross-correlation power spectrum between the two images:

(1) $$R\left(u\right)=\frac{F\left(u\right)G^{*}\left(u\right)}{\left|F\left(u\right)G^{*}\left(u\right)\right|}=exp\left[-i2\pi \left(\frac{ux_{0}}{M}\right)\right]\;$$

where * indicates the complex conjugate, R(u) is the cross-correlation function in the frequency domain, and *M* is the size of the image in the measurement dimension. Finally, R(u) obtains a one-dimensional δ function representing the number of pixels that shifted between images through inverse transformation:

(2) $$r\left(x\right)=IFT\left[R\left(u\right)\right]=\delta \left(x-x_{0}\right)$$

The second step was sub-pixel positioning. The aim of this step was to define the peak position in the spatial domain and then use the sinc function to achieve interpolation during the inverse transformation, under the condition that the power spectrum remains unchanged.

After obtaining the position of the whole-pixel $x_{0}$ in the first step, an area of 1.5 pixels size centered at this position was taken, and the normalized inverse Fourier transform of *k* times up-sampling on the power spectrum function R(U) was performed to obtain the interpolated one-dimensional δ function $r\left(X^{\prime }\right)$, as shown in Equation (3):

(3) $$r\left(X^{\prime }\right)=\frac{1}{MK}e^{\left(i2\pi \frac{X^{\prime }}{k}\frac{U^{T}}{M}\right)}R\left(U\right)$$

where $X^{\prime }\;$=${\left[0,\;1,\ldots ,\;1.5k-1\right]}^{T}-\frac{1.5k}{2}+X_{0}k$, $U\;=\;{\left[0,\;1,\ldots ,\;M-1\right]}^{T}-M/2$. Finally, the number of translation pixels with sub-pixel accuracy was obtained.

### 2.3. Design of the Moving Target and Realization of Large Range

**Figure 2:**
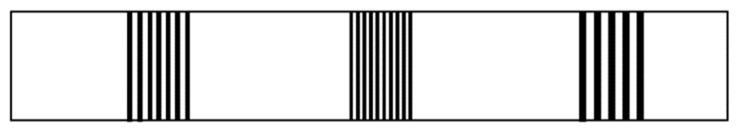
Multiple sets of stripe patterns with different intervals.

In order to increase the range of the image grating measurement system, multiple sets of stripe patterns with different intervals were designed on the glass plate as moving targets, as shown in Figure 2. The LED light is passed through the glass plate to the line scan camera, and a high-contrast image of the stripe can be taken, thereby effectively preventing the influence of noise.

In order to avoid confusion in registration, the uniqueness of each section was considered. Specifically, two requirements were followed: (1) the characteristics of at least three groups of adjacent stripe sections should be visually different; and (2) two adjacent sections should be captured in one FoV but sufficiently cover the FoV to minimize the overlaps.

Table 1 is a series of images captured by the camera when the stage was at 8 consecutive different positions during the measurement process. There was a complete set of stripes in each FoV to match the stripe pattern at the previously selected reference position. In addition, the next set of stripes appeared before the first set of stripes moved out. Such a continuous pattern matching methodology was applied to expand the measurement range beyond a single FoV.

The design of the patterns was based on the DFT local up-sampling phase correlation method, so the periodicity of the discrete Fourier transform must limit the sampling interval between the stripe images. The periodicity of the initial image g(x) in the frequency domain can be expressed as:

(4) G(u)=G(u+kM)

where *M* is the size of the image and *k* is an integer. g(x) was shifted to the right by $\frac{\left(M+1\right)}{2}$ pixels, as shown in Equation (5):

(5) $$G\left(u-\frac{M+1}{2}\right)=G\left[u+kM-\frac{M+1}{2}\right]=G\left[u+\left(k-1\right)M+\frac{M-1}{2}\right]$$

It can be seen from Equation (5) that the image was translated to the right by $\frac{M+1}{2}$ pixels, and after the DFT, it was translated to the left by $\frac{M-1}{2}$ pixels. In other words, when the stripe pattern was translated by more than half the size of the camera’s FoV, the difference between the size of the FoV and the actual displacement was obtained. Therefore, the sampling interval between the initial image g(x) and the translated image f(x) cannot exceed half the size of the camera’s FoV.

In addition, the sampling interval was also dependent on the initial position of the stripe pattern in the FoV, because the set of patterns cannot be outside the FoV after the translation.

**Figure 3:**
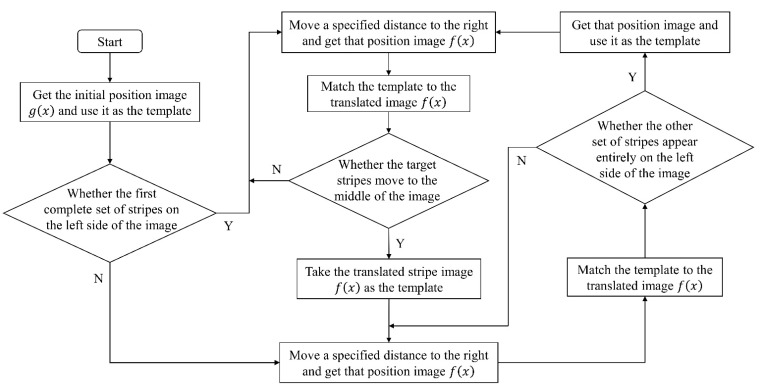
Flowchart of displacement measurement and measurement range expansion.

In summary, a smaller sampling interval was selected in this study, and the process of displacement measurement and measurement range expansion can be expressed by the flowchart in Figure 3.

This is a displacement measurement method that can achieve a low sampling frequency. The sampling frequency can be set reasonably according to the desired position information, which avoids the phenomenon whereby the computer is overloaded with too many collected images. At the same time, if the information collection fails at a certain position, the subsequent displacement can still be obtained normally, which improves the robustness of the system. The stitching of the measurement range in the system depends on the switching of the template. The switching of the template must be performed every time half of the FoV is crossed, which means that the accumulation of the range is required only after the displacement of half the FoV is completed. Therefore, the image grating system does not have excessive accumulated error compared with traditional grating sensors.

### 2.4. Fast Calibration Method Based on Pinhole Imaging Model

**Figure 4:**
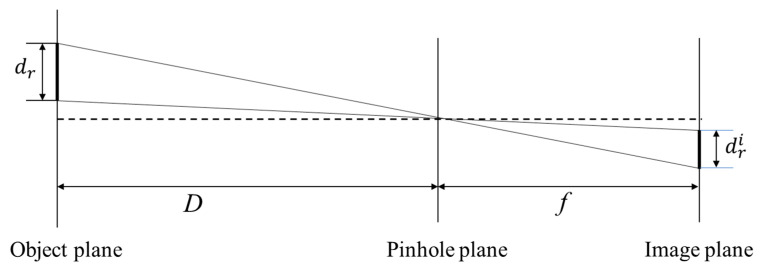
Pinhole imaging model with the optical axis perpendicular to the object plane.

The image correlation algorithm only calculates the number of translation pixels between the images. To obtain the displacement of the object in the physical space, it is necessary to establish the corresponding relationship of the object in the image space. The fast calibration method [26] based on the pinhole imaging model is a calibration method that does not require one-by-one conversion of the coordinate system and it can quickly and accurately obtain the pixel equivalent, the ratio of the size of the object to the number of pixels it occupies on the imaging sensor. The pinhole imaging model with the optical axis perpendicular to the object plane is shown in Figure 4.

According to the principle of perspective projection, the pixel equivalent can be expressed as:

(6) $$s=\frac{d_{r}}{I_{r}}\;\mathrm{or}\;s=\frac{d_{r}}{d_{r}^{i}}d_{pixel}=\frac{D}{f}d_{pixel}$$

where s is the pixel equivalent, $d_{r}$ is the actual size of the object involved in imaging, $I_{r}$ is the number of pixels the object occupies on the image plane, $d_{r}^{i}$ is the actual physical size of the object on the image plane, $d_{pixel}$ is the size of each pixel, $d_{r}^{i}=I_{r}\cdot d_{pixel}$, f is the focal length, and D is the distance from the object plane to the focal point.

## 3. System Setup

When building the system, a uniformly luminous LED backlight was selected, which effectively inhibits the influence of light intensity variation on the pixel equivalent. A line scan camera was used as the displacement reader. Its smaller pixel size ensured the accuracy of the displacement calculation. Comparing the system proposed in this paper with the previous system developed by the authors’ team, the most obvious differences are:

(1) it is equipped with an ultra-low distortion lens, which can minimize the errors induced by image matching and optical magnification;
(2) multiple groups of target stripes with different characteristics are designed on the glass plate, which can expand the range in combination with the image matching algorithm.

**Figure 5:**
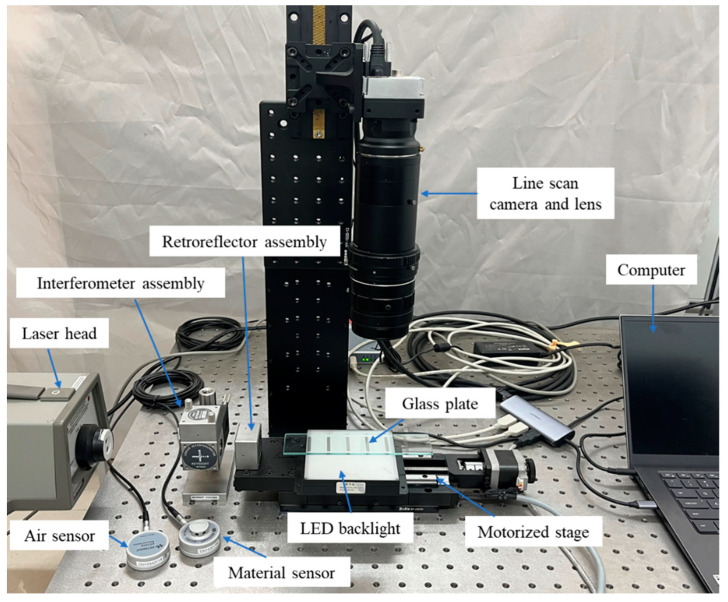
The experimental system setup.

Information regarding the main hardware is shown in Table 2 below, and the experimental system setup is shown in Figure 5. In this project, six groups of stripes were fabricated on the glass plate. The length of each group is 3.6 mm, and the interval distance is 18 mm. In the first three groups, the length of the line pair is 120 μm, 90 μm, and 150 μm, respectively, and the latter three groups repeated the same design as a back-up. In the experiments of this presented work, three groups of stripes were used.

**Figure 6:**
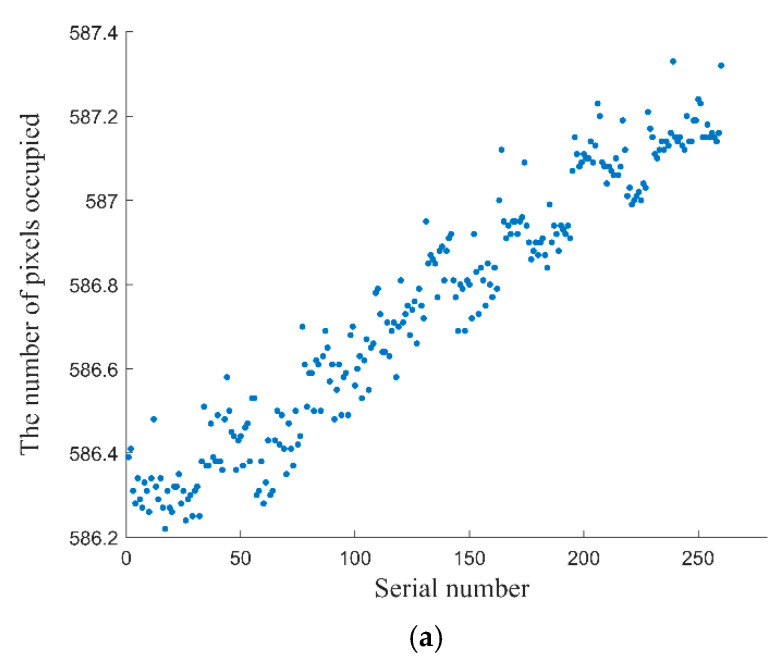

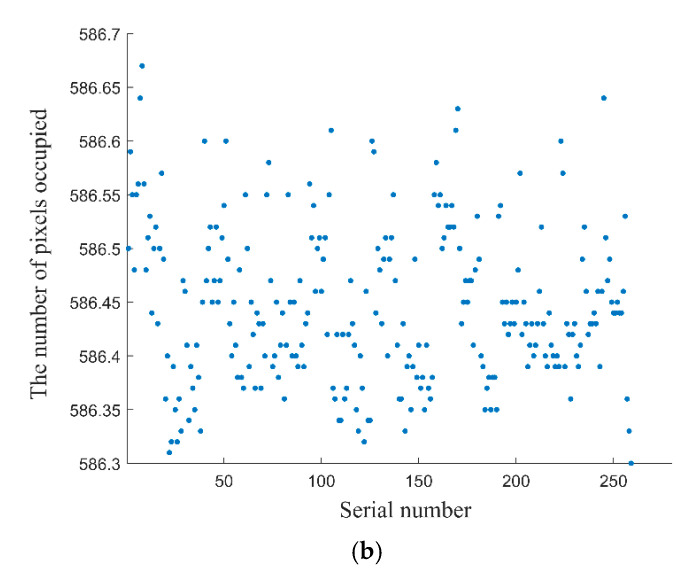
Variation in the number of pixels when the optical axis of the camera is tilted and perpendicular. (**a**) Variation in the number of pixels when the optical axis is tilted. (**b**) Variation in the number of pixels when the optical axis is perpendicular to the glass plate.

Theoretically, the magnification will only stay consistent when the optical axis is exactly perpendicular to the glass plate [27]. In order to avoid the perspective error caused by the tilt of the optical axis, a glass plate filled with stripes with a line pair width of 100 μm was placed in the FoV to adjust the perpendicularity of the optical axis. The adjustment was performed until the number of pixels occupied by the 20 line-pair stripes at each position in the FoV remained consistent. Figure 6 shows the pixel occupation before and after the perpendicularity adjustment. When the optical axis is tilted, the number of pixels occupied by every 20 line-pair stripes at each position in the FoV shows a linear trend, as shown in Figure 6a. After a proper adjustment, the pixel number occupied by the same stripe set remained approximately consistent.

## 4. Error Analysis of the Measurement System

### 4.1. Length Error of Each Group of Stripes

**Figure 7:**
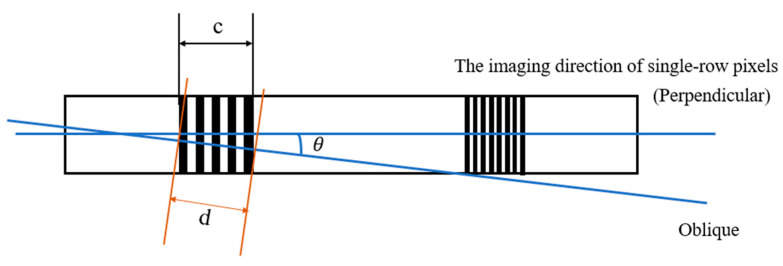
Schematic diagram of the misalignment of the imaging axis.

Since the pixel equivalent was the ratio of the stripe length to the number of pixels occupied, the measurement deviation in the stripe length directly affected the accuracy of the pixel equivalent, which in turn caused linear systematic errors in the proposed measurement system. There were two factors that produced this deviation: (1) the dimension bias against the nominal value; and (2) the misalignment of the imaging axis, that is, the stripes are not completely perpendicular to the imaging direction of the single-row pixels of the line scan camera, as shown in Figure 7.

The resulting error is shown in Equation (7),

(7) e=d−c=c(1/cosθ−1) 

where d=c/cosθ. It can be calculated that a misalignment of 0.5° will induce a calibration error of 0.14 μm for a 3.6 mm stripe set.

**Figure 8:**
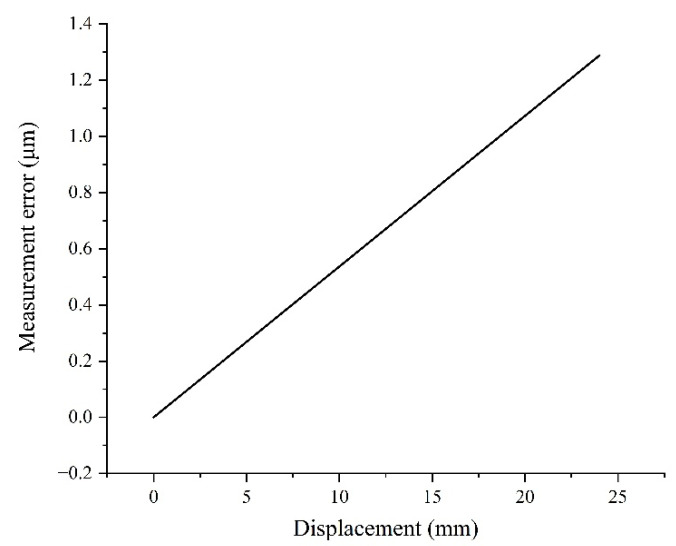
Displacement measurement error curve caused by stripe length error.

Assuming that the length error of the 3.6 mm stripes used in the calculation of pixel equivalent is 0.2 μm, the displacement measurement error generated will linearly increase to 1.3 μm within the range of 24 mm at an optical magnification close to 1:1, as shown in Figure 8.

### 4.2. Optical Distortion

The camera lens may have pincushion or barrel distortion due to manufacturing and assembly reasons. Although an ultra-low distortion lens was employed, it is still necessary to explore the effects of distortion under sub-micron measurement accuracy. Because the line scan camera had only one imaging unit in one direction, when only radial distortion is considered, the pixel equivalent s(i) of uneven magnification can be represented by a fourth-order polynomial [23,28]:

(8) $$s\left(i\right)=a+bx^{2}\left(i\right)+cx^{4}\left(i\right)\;$$

At this time, the calculation of displacement x is shown in Equation (9),

(9) $$x=\int_{i_{0}}^{i_{t}}s\left(i\right)di\;\;$$

where $i_{0}$ and $i_{t}$ are the start and end positions of the pixel index, respectively.

After discretization, the displacement measurement error caused by optical distortion can be expressed as:

(10) $$E=\sum_{i_{0}}^{i_{t}}s\left(i\right)-\bar{s}\left(i_{t}-i_{0}\right)$$

where $\bar{s}$ is the pixel equivalent under the undistorted center of the FoV, and E is the measurement error caused from $i_{0}$ to $i_{t}$.

**Figure 9:**
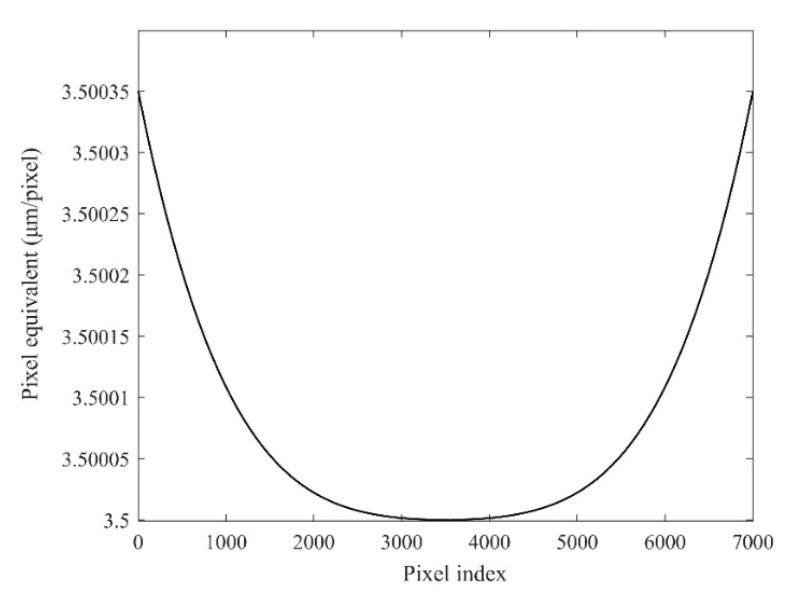
The pixel equivalent at different positions in the FoV.

**Figure 10:**
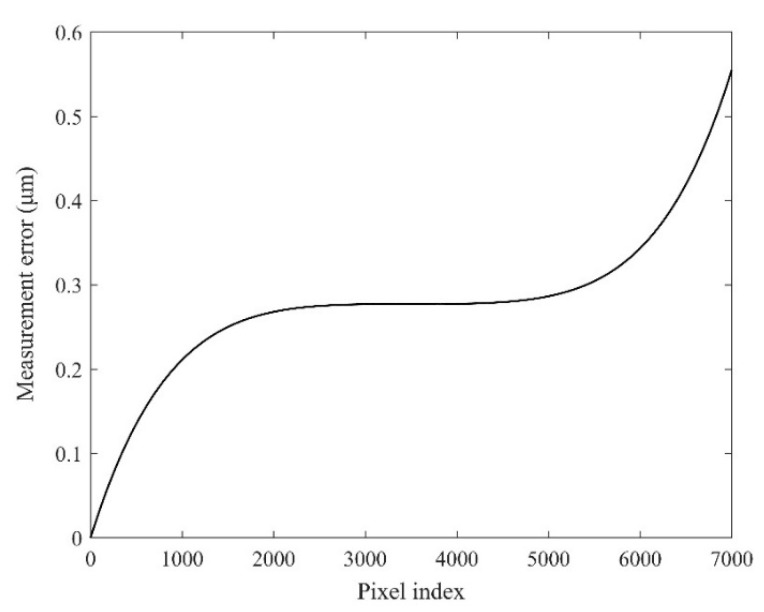
Displacement measurement error curve in the full FoV.

Table 2 shows that the distortion of the lens is 0.01%, the magnification is 1×, and the pixel size is 3.5 μm. Assuming that the lens has barrel distortion, then the pixel equivalent of the edge area of the image from the relative distortion of 0.01% can be expressed as s(0) = s(7000) = 3.50035 μm/pixel. Meanwhile, according to the variation trend in the barrel distortion, the pixel equivalent at the 1/4 area can be obtained as s(1750) = s(5250) = 3.500035 μm/pixel. Because the image center is an undistorted area, the pixel equivalent there can be represented as $\bar{s}$ = s(3500) = 3.5 μm/pixel. The pixel index in the FoV is 0 to 7000, so the pixel equivalent of the lens at different positions in the FoV is shown in Figure 9, and the displacement measurement error in the full FoV is close to 0.55 μm, as shown in Figure 10.

### 4.3. Motion Error in the Direction of the Optical Axis

**Figure 11:**
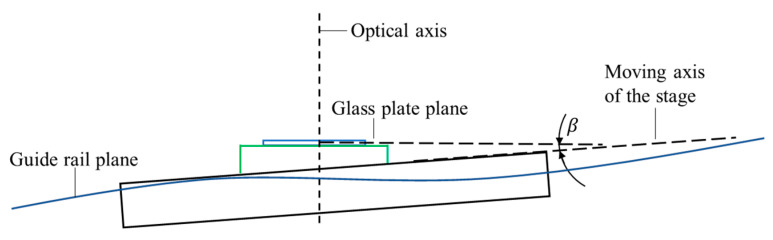
Schematic diagram of motion error in the direction of the optical axis.

In the proposed image grating system, although the perpendicularity between the optical axis and glass plate has been optimized, the straightness of the guide rail and the non-parallelism between the glass plate and the moving axis of the stage will still cause the motion error in the target stripes on the glass plate in the optical axis of the camera, as shown in Figure 11. The stand-off distance will vary with the movement of the stage. Therefore, in the case of using a non-telecentric lens, it is necessary to study the influence of the stand-off variation on the pixel equivalent of the measurement system and the displacement measurement error induced.

**Figure 12:**
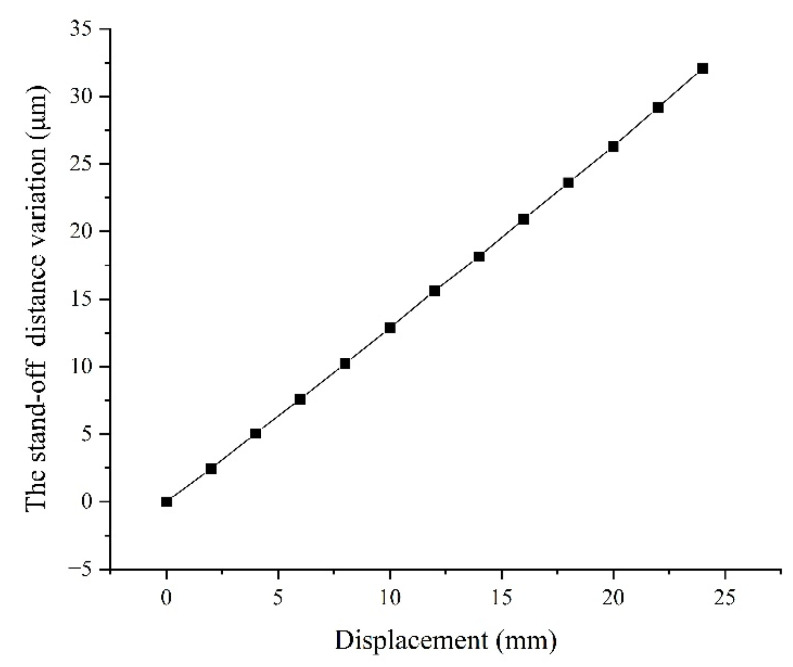
The stand-off distance variation in the target stripes in a FoV.

In order to more accurately measure the stand-off distance variation in the target stripes during the movement, a chromatic confocal sensor (Micro-Epsilon, IFS2406-2,5/VAC) was installed in the direction of the optical axis. The results of the distance variation within a FoV are shown in Figure 12. It can be seen that the stand-off varied from about 0 to 32 μm during the movement of the stage to 24 mm, and presented an increasing linear trend.

**Figure 13:**
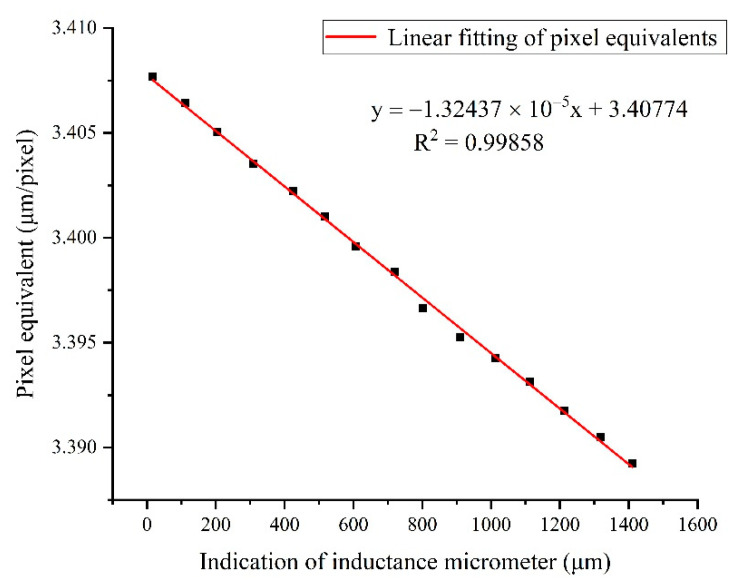
Change curve of pixel equivalent with stand-off distance.

From Equation (6) in Section 2.4, we can see that the pixel equivalent $s=\frac{D}{f}\cdot d_{pixel}$, where $d_{pixel}$ and f are constants; thus, the pixel equivalent s has a linear relationship with the stand-off distance D of the target stripes. Therefore, a corresponding experiment was performed to calibrate the linear coefficient. First, a set of stripe patterns on the glass plate used for measuring was placed in the center of the FoV, and then the positions of the camera and lens were adjusted up and down near the depth of field. The distance of each position was measured by the inductance micrometer (TESA-TT80), and the number of pixels occupied by the stripe pattern at the corresponding position was counted, and the relationship curve between the pixel equivalent and the stand-off distance was obtained, as shown in Figure 13. The slope of the fitted straight line in the figure is the linear coefficient to be calibrated.

**Figure 14:**
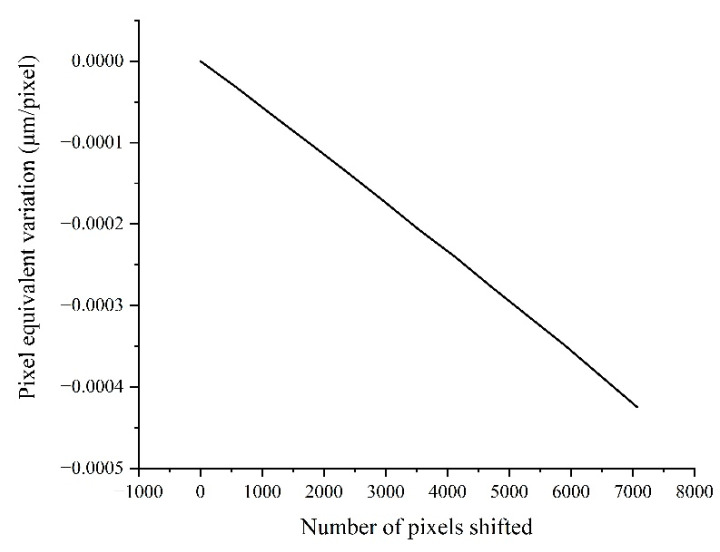
The pixel equivalent variation caused by the varying stand-off distance.

**Figure 15:**
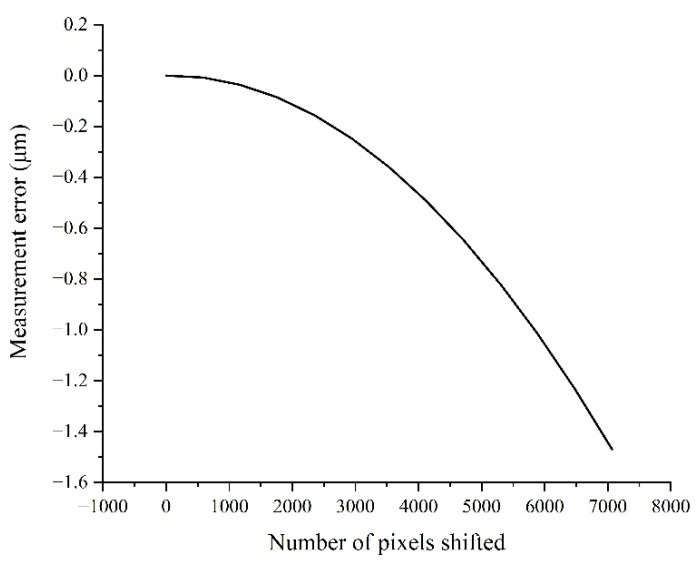
The measurement error caused by the varying stand-off distance.

Combined with the stand-off distance variation shown in Figure 12, the pixel equivalent variation in the FoV can be obtained, as shown in Figure 14. Then, the measurement error induced was obtained from Equation (9), as shown in Figure 15. With the increasing number of shifted pixels, the displacement measurement error reached about 1.5 μm. Therefore, it was inferred that the motion error of the target stripes in the direction of the optical axis had a significant impact on the displacement measurement.

## 5. Experiment and Result Analysis

### 5.1. Preliminary Experiment and Result Analysis

First, a glass plate filled with stripes with a line pair width of 100 μm was used for calibration to obtain the mean value of pixel equivalent at different positions in the FoV. The line pair at each location was taken to calculate the pixel equivalent. Then the average pixel equivalent was used in the calculation of the displacement.

In order to verify the measurement capability of the proposed system, repeated experiments were performed. In the experiment, an Agilent 5530 laser interferometer was used as the reference instrument for displacement measurement. The temperature in the air-conditioned laboratory varied from 22.51 °C to 22.88 °C. In the experiment, NI software LabVIEW was used to control the movement of the stage and the acquisition of images. During the measurement, stripe images and reference displacements were collected every 1 mm. At the same time, due to the size of the LED, only three of the stripes on the glass plate were involved in the measurement. After the measurement was completed, the up-sampling factor *k* = 100 was taken for image registration and measurement displacement calculation.

**Figure 16:**
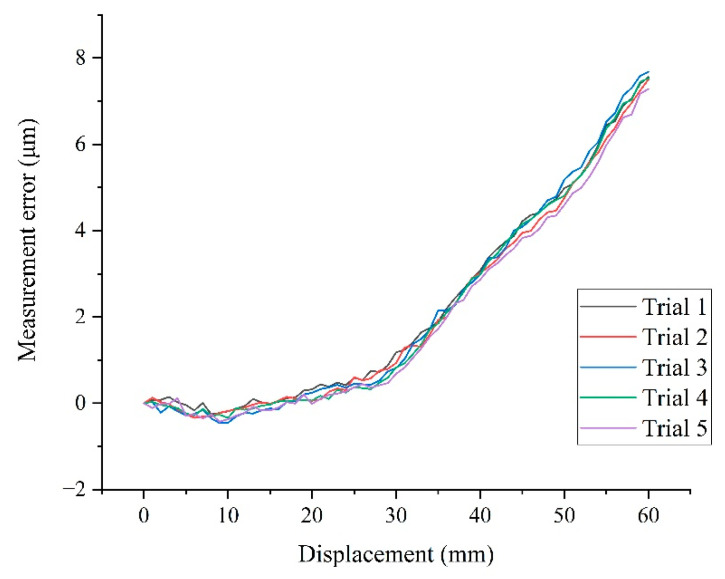
Five repeated measurement errors calculated by the mean value of pixel equivalent.

The preliminary experimental results were obtained from five repeated measurements within 35 min, as shown in Figure 16. It can be seen that with the stitching of the FoVs, where the three sets of stripes were located, the measurement range of the image grating system reached 60 mm. The overall error in this range was between −0.5 and 7.5 μm, and it showed a non-linear trend due to the influence of the pixel equivalent variation in the measurement. It can be concluded from Section 4.2 and Section 4.3 that the influence of motion error in the optical axis direction on the pixel equivalent was more significant than that of the optical distortion. When a fixed average pixel equivalent was taken into account, but the actual pixel equivalent varied during the stage movement, measurement errors would occur. Therefore, the measurement error, which was mainly due to the axial fluctuation, needed dynamic calibration during the stage movement.

### 5.2. Dynamic Calibration of Pixel Equivalent

Based on the discussion in Section 4 and the previous section, the optical distortion of the lens and the motion error in the optical axis direction will significantly affect the pixel equivalent, and therefore they are key error factors. Since it is difficult to separate these two factors and the error translation models will change when the system is re-installed, it is necessary to dynamically calibrate the pixel equivalent for error compensation purpose.

**Figure 17:**
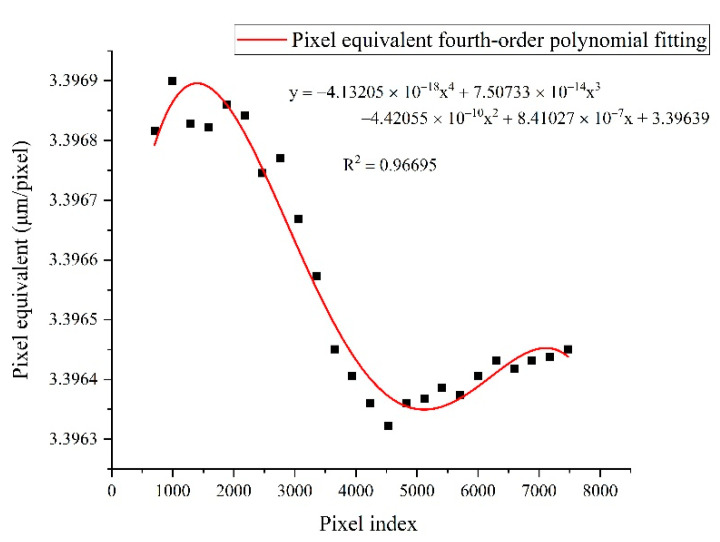
The dynamically varying pixel equivalent in the FoV.

In order to dynamically determine the pixel equivalent, the glass plate with patterned features used for measuring displacement was also used as a tool to calibrate the pixel equivalent. As shown in Figure 2, the patterned features include multiple sets of stripes with the same width and different line pairs. A certain set of stripes on the glass plate was placed at the far left of the camera’s FoV, and then stripe images were collected every 1 mm as the stage was moving rightwards until the set of stripes moved out of the FoV. By recording the pixel numbers occupied by this set of stripes at different positions in the FoV, the pixel equivalent variation in the FoV can be determined, as shown in Figure 17.

Then, the calibration of the pixel equivalent s can be accurately achieved by using fourth-order polynomial fitting, and s(i) is the expression of the dynamically changing pixel equivalent in the FoV. Therefore, the actual displacement of the stripe pattern can be accurately obtained from Equation (9).

### 5.3. Error Compensation and Analysis

**Figure 18:**
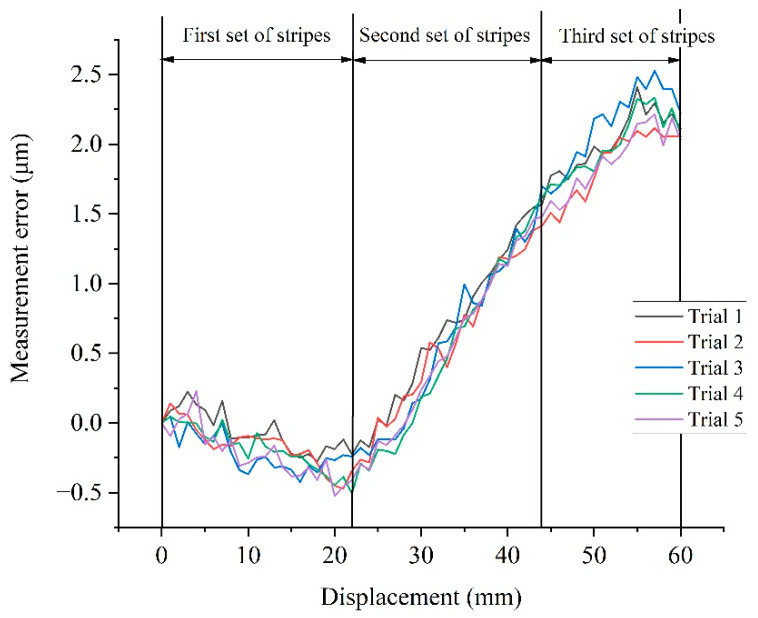
Five repeated measurement errors after dynamic calibration of pixel equivalent.

After the dynamic calibration of the pixel equivalent, the final experimental results were obtained from five repeated measurements in Section 5.1, as shown in Figure 18. It can be easily seen that the measurement range was expanded by stitching the FoV traversed by the three stripe sets. The error of the proposed measurement system was better than 2.5 μm within the measurement range of 60 mm. Compared with the measurement results shown in Figure 16, the dynamic calibration of the pixel equivalent significantly reduced the measurement error of the proposed system and eliminated the nonlinear errors in the system. Its standard deviation, which can be used as an indicator of measurement repeatability, was calculated to be less than 0.16 μm in this travel, as shown in Table 3.

Figure 18 also shows that the displacement area of each group of stripes had apparent linear systematic error, which was determined by the length error of each group of stripes. Different slopes shown in different sections indicate the calibration uncertainty for the length of each stripe set. With the current referencing technology, the measurement uncertainty of the stripes remained at submicron level. As a result, in a measurement range of 20 mm, which was covered in each stripe set, this reference-induced error increased with the displacement and reached around 1 micron.

The results showed that although the calibration error played a significant role in the overall measurement accuracy, the proposed system is able to perform highly repeatable measurements in a long travel range.

## 6. Conclusions

In this study, an improved image grating system for displacement measurement was developed, as a continuation of the earlier work published by the authors’ team [23]. It featured an ultra-low distortion lens, measurement range expansion algorithm and pixel equivalent dynamic calibration method. Based on the DFT local up-sampling phase correlation method, sub-pixel interpolation and multi-FoV stitching were achieved. Therefore, the proposed system was able to perform high-resolution measurement over a long range. In addition, the main error factors were identified to be optical distortion, the motion error of the stage (linearity error that varies the stand-off distance) and calibration uncertainty of the stripes on the target glass plate.

With the dynamic calibration of the pixel equivalent, the nonlinear errors due to optical distortion and the motion error can be well compensated for. Experimental results showed that this dynamic calibration method was able to reduce the measurement error from 7.5 μm to 2.5 μm, in a measurement range of 60 mm. The proposed system also showed a respectable measurement repeatability of 0.16 μm in the measurement range of 60 mm, and this repeatability is purely dependent on the image correlation algorithm. Therefore, the repeatability will not be worse if the measurement range is further expanded.

After the error compensation based on dynamic calibration, the system still showed a residual measurement error of 2.5 μm in a range of 60 mm, and the measurement error in each section showed a linear trend. This was due to the calibration error in the length of each set of stripes. The calibration of the stripes’ length typically has an uncertainty of 0.1–0.3 μm. In our measurement setup, a measurement error of up to 2 μm will be introduced.

Compared with the authors’ earlier work, which is presented in [23], the methodology proposed in this paper to expand the measurement range has introduced measurement errors, which are not as correctable as the earlier published work. In [23], the measurement was performed in one FoV only. After compensating for the optical distortion, there was only a linear error, which can be easily compensated for physically or analytically. However, in the expanded measurement range, by combining multiple FoVs each FoV showed linearly increasing or decreasing measurement errors, due to stripe size calibration uncertainty, as discussed in Section 5.3. As a result, the measurement errors did not show consistent linearity in the whole range. This nonmonotonicity exposed an important error source that requires further study by the author. Developing a higher-accuracy calibration method, will be the next step of this project.

## Figures and Tables

**Table 1:** Stripe images at different positions.

Position Number	Stripe Images	Position Number	Stripe Images
(1)	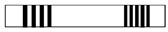	(5)	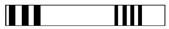
(2)	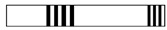	(6)	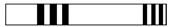
(3)	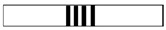	(7)	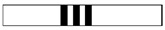
(4)	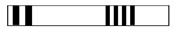	(8)	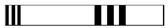

**Table 2:** The main hardware in the proposed system.

Hardware	Models/Manufacturers	Technical Specifications
Camera	ral8192-12gm-Basler racer	Resolution (H × V): 8192 px × 1 px Sensor: DR-8k-3.5
Optical lens	OPT-VHK120/5.8-1.0×	Focal length: 120 mm Magnification: 1.0× Chart size: 63 mm Distortion: 0.01%
Glass plate	Hong Cheng Optical Products, Dongguan, China	Manufacturing tolerance of the stripes: ±1 μm
Motorized stage	Zolix-CXPF100-80175	Stroke: 100 mm Positioning repeatability: ±2 μm Straightness: ≤10 μm

**Table 3:** Measurement deviation of the proposed system.

Displacement (mm)	3	8	13	18	23	28	33	38	43	48	53	58
Error mean (μm)	0.07	0.15	0.16	0.33	0.23	0.04	0.53	1.03	1.46	1.81	2.06	2.14
Standard deviation (μm)	0.09	0.04	0.12	0.05	0.08	0.12	0.13	0.04	0.07	0.10	0.15	0.16

## Data Availability

Data available in a publicly accessible repository.
